# Using Twitter to Examine Stigma Against People With Dementia During COVID-19: Infodemiology Study

**DOI:** 10.2196/35677

**Published:** 2022-03-31

**Authors:** Juanita-Dawne Bacsu, Sarah Fraser, Alison L Chasteen, Allison Cammer, Karl S Grewal, Lauren E Bechard, Jennifer Bethell, Shoshana Green, Katherine S McGilton, Debra Morgan, Hannah M O’Rourke, Lisa Poole, Raymond J Spiteri, Megan E O'Connell

**Affiliations:** 1 Department of Psychology Canadian Centre for Health and Safety in Agriculture University of Saskatchewan Saskatoon, SK Canada; 2 Interdisciplinary School of Health Sciences Faculty of Health Sciences University of Ottawa Ottawa, ON Canada; 3 Department of Psychology University of Toronto Toronto, ON Canada; 4 College of Pharmacy and Nutrition University of Saskatchewan Saskatoon, SK Canada; 5 Department of Kinesiology and Health Sciences Faculty of Health University of Waterloo Waterloo, ON Canada; 6 Knowledge, Innovation, Talent and Everywhere (KITE) - Toronto Rehabilitation Institute University Health Network Toronto, ON Canada; 7 Canadian Centre for Health and Safety in Agriculture University of Saskatchewan Saskatoon, SK Canada; 8 Faculty of Nursing University of Alberta Edmonton, AB Canada; 9 Dementia Advocacy Canada Calgary, AB Canada; 10 Department of Computer Science University of Saskatchewan Saskatoon, SK Canada

**Keywords:** coronavirus 2019, social media, stigma, dementia, ageism, COVID-19, Twitter, bias, infodemiology, attention, risk, impact, misinformation, belief, cognition, cognitive impairment

## Abstract

**Background:**

During the pandemic, there has been significant social media attention focused on the increased COVID-19 risks and impacts for people with dementia and their care partners. However, these messages can perpetuate misconceptions, false information, and stigma.

**Objective:**

This study used Twitter data to understand stigma against people with dementia propagated during the COVID-19 pandemic.

**Methods:**

We collected 1743 stigma-related tweets using the GetOldTweets application in Python from February 15 to September 7, 2020. Thematic analysis was used to analyze the tweets.

**Results:**

Based on our analysis, 4 main themes were identified: (1) ageism and devaluing the lives of people with dementia, (2) misinformation and false beliefs about dementia and COVID-19, (3) dementia used as an insult for political ridicule, and (4) challenging stigma against dementia. Social media has been used to spread stigma, but it can also be used to challenge negative beliefs, stereotypes, and false information.

**Conclusions:**

Dementia education and awareness campaigns are urgently needed on social media to address COVID-19-related stigma. When stigmatizing discourse on dementia is widely shared and consumed amongst the public, it has public health implications. How we talk about dementia shapes how policymakers, clinicians, and the public value the lives of people with dementia. Stigma perpetuates misinformation, pejorative language, and patronizing attitudes that can lead to discriminatory actions, such as the limited provision of lifesaving supports and health services for people with dementia during the pandemic. COVID-19 policies and public health messages should focus on precautions and preventive measures rather than labeling specific population groups.

## Introduction

The COVID-19 pandemic is taking a serious toll on people with dementia and their care partners. In Canada, dementia is the most common comorbidity, accounting for 36% of all COVID-19-related deaths [1]. Increased age, medical frailty, and health conditions often associated with dementia increase the risk for complications from COVID-19 [2,3]. Consequently, increased COVID-19 risk and vulnerability for people with dementia have been emphasized by governments, health care clinicians, the general public, and traditional news media (eg, radio and television news, print media, and affiliated websites) using a 1-to-many communication structure (ie, 1 verified individual or group is the publisher of information for many consumers) [4-7].

Beyond dissemination of information by traditional news media outlets, Twitter and other forms of social media (eg, Facebook, YouTube, Instagram) have been used extensively by individuals during the pandemic in a many-to-many communication structure (ie, any individual can be both a publisher and a consumer of information) to share messages about COVID-19 [7,8]. Social media is generally described as internet-based channels of mass personal communication fostering perceptions of interactions among users, deriving value predominantly from user-generated material and content [9]. For example, people have been using Twitter to share opinions, fears, and beliefs regarding the COVID-19 pandemic. However, a consequence of this many-to-many communication structure is that any member of the network can share inconsistent, contradictory, or even false information about the pandemic. Accordingly, this messaging can lead to COVID-19 misconceptions, false information, and stigma (eg, stereotypes, negative beliefs, and discriminatory behavior) toward individuals or groups that are often already marginalized [10,11].

Recently, an emerging body of literature has identified COVID-19-related stigma and ageism against older adults in social media discourse [12,13]. Ageism reflects how we think, feel, and act toward others or oneself based on age [14]. The model of stigma communication [15,16] identifies 4 types of message content (mark, group labeling, responsibility, and peril): (1) a mark to classify people in a stigmatized group, (2) descriptions or labels of the stigmatized group as a separate entity from the rest of society (eg, us vs them), (3) assigning responsibility for placement in the stigmatized group, and (4) cues to imply that the stigmatized group is a peril or threat to society that needs to be addressed through collective efforts. In 2021, a study analyzing 536 tweets under the #BoomerRemover hashtag reported issues of intergenerational conflict, ageism, and stigma toward older adults during the pandemic [17]. Another study examining 351 tweets about older adults during the COVID-19 pandemic found that almost 88 (25%) of the tweets had stigma, ageism, or potentially offensive content toward older adults [18]. Stigma against older adults is not limited to the pandemic. In 2017, Oscar et al [19] analyzed tweets on Alzheimer disease and reported that 21% of the tweets fostered stereotypes and stigma.

Addressing stigma against dementia on social media is important because it can have severe implications, including depression, anxiety, fear, social isolation, feelings of shame, and a decreased quality of life for people with dementia and their care partners [20-25]. Research shows that stigma against dementia can negatively impact interactions with health care providers, experiences in acute care settings, and access to specialist services and delay a timely dementia diagnosis [24,25]. However, a timely diagnosis enables people with dementia to acquire support services, obtain relevant information, plan for the future, and access pharmaceutical treatments that may improve their cognition and quality of life [21].

Apart from the 1 study by Oscar et al [19], there is a paucity of research exploring social media discourse related to stigma against dementia, especially in the context of the COVID-19 pandemic. Furthermore, the unknown effects of COVID-19 may perpetuate fear, blame, and false beliefs, leading to increased stigmatization against marginalized groups, especially for groups deemed as being more at risk to the virus, such as people with dementia. Examining how stigma manifested on social media during the pandemic may deepen our understanding of the methods and content used to facilitate stigma communication against people with dementia and their care partners.

With over 330 million monthly users [26], Twitter presents a novel opportunity to expand the repertoire of qualitative research approaches and data collection methods [27] by using a comprehensive, publicly available data set for infodemiology. Infodemiology (ie, information epidemiology) is defined by Eysenbach as the study of the distribution and determinants of health information on the internet to inform health professionals and health policy [28]. Given the high volume of active users, Twitter provides an innovative means for understanding different COVID-19 perspectives that may have been ignored or concealed due to the current challenges of conducting in-person research during the pandemic. Using Twitter data, the objective of this study was to examine social media discourse on stigma against people with dementia during the pandemic.

## Methods

### Ethics

Drawing on existing Twitter studies, there is a general consensus that tweets can be used for research without requiring ethical approval, because analyzing publicly available text on social media platforms, such as Twitter, is generally not considered human subject research [18,19]. Because tweets posted on the Twitter website are located within the public domain, ethics approval was not obtained. However, we removed any identifying information related to usernames or handles (eg, @name) to help protect the anonymity of the Twitter users.

### Recruitment

Tweets were collected on Twitter using the GetOldTweets application in Python from February 15 to September 7, 2020. Search terms for the tweets included dementia or Alzheimer disease used in combination with COVID-19, coronavirus, or COVID, resulting in 20,800 tweets. Nonoriginal tweets and retweets were excluded from the study. Filters were applied to exclude irrelevant tweets (eg, spam, advertising), resulting in 5063 (24.34%) tweets. The 5063 tweets were analyzed by a group of coders [29], with 1743 (34.43%) tweets identified for stigma-related coding. Given that the tone of the tweets can be difficult to interpret, our process for identifying stigma-related tweets for inclusion was broadly based in attempts not to overlook any relevant tweets. Specifically, our inclusion criteria included tweets that perpetuated stigma (eg, political dementia–related insults, assigning blame to people with dementia, self-stigma, stereotypes or labeling people with dementia, and misinformation about dementia), tweets that devalued the lives of older adults or people with dementia, and tweets that challenged stigma against people with dementia. The 1743 tweets were extracted into a Microsoft Excel spreadsheet for data analysis. An Excel spreadsheet was used to support ease of use among the large research team because it did not require additional training in order to use the software.

### Data Analysis

The 1743 tweets were analyzed using thematic analysis after line-by-line coding, a qualitative method that identifies key topics and patterns in the data, with the objective of identifying the overarching themes [30]. To develop a robust codebook, 4 researchers (authors JDB, MEO, SF, ALC) read and re-read 100 (4.74%) tweets multiple times to become immersed within the Twitter data and develop the initial codes. The stigma communication model [15,16] was used in the development of our initial codebook by including codes such as the mark (eg, a mark to classify people in a stigmatized group), responsibility (eg, assigning blame by making attributions about the lifestyle or actions of people with dementia), peril (eg, suggesting that people with dementia are a social, physical, or economic threat to society), and group labeling (eg, descriptions or labels of people with dementia as separate from the rest of society). The codebook contained definitions, cues, and tweet examples for each of the codes. Pilot tests were conducted to test intercoder consistency with the researchers independently coding the same tweets and then meeting to compare coding and further revise the codebook (eg, deleting or adding additional codes for emerging themes). After multiple meetings, the codebook was further refined by merging or deleting any overlapping or unused codes. The final version of the codebook consisted of 6 codes: (1) devaluing the lives of older adults (eg, “old and dying anyways”), (2) responsibility/blame (eg, “dementia COVID economy”), (3) false information (eg, COVID-19 vaccine causes dementia), (4) political dementia–related stigma (eg, “dementia Joe”), (5) self-stigma (eg, “don’t save me, save my grandchildren”), and (6) challenging stigma against dementia (eg, “it’s a scandal how dementia patients are treated”).

Once the codebook was completed, the researchers met with the coding team. The coding team consisted of 12 coauthors, who received 4.5 hours of coding training (eg, individual practice coding exercises, collaborative team coding to identify and address any coding challenges or questions, team meetings to discuss specific coding questions, and partner coding activities). After the coding training was completed, the 1743 tweets were divided among the 12 coders. Each coder received 268 (15.38%) tweets, which allowed for each tweet to be independently coded by 2 different coders to ensure intercoder reliability. Any coding challenges or discrepancies were resolved through collaborative discussion and consensus.

After coding was completed, team meetings were held to conduct thematic analysis [30] by examining the primary themes and subthemes that emerged from reviewing the researchers’ reflexive memos [31] and re-reading the tweets under each of the codes. All the researchers were experienced in thematic analysis, and 1 trainee (author KSG) was mentored by working in direct collaboration with the research team. Reflexivity was used to recognize the researchers’ positionality in terms of their own judgments and beliefs to help ensure that these did not influence the analysis [30]. An additional team meeting was held to reach group consensus on the overarching themes and to identify exemplar tweets (eg, accessible, no acronyms) for publication.

### Rigor

Three measures were used to ensure rigor in our research. First, the research team used reflexive memos to record notes about emerging patterns, themes, and relationships during the coding process [31]. Second, multiple coding (eg, each tweet coded independently by 2 coders) was used to provide cross-checks in the interpretation of the tweets by independent researchers [32]. Moreover, having each tweet coded independently by 2 reviewers helped to ensure intercoder reliability, with an average of 86% agreement between the coders. The intercoder reliability was determined by calculating each percentage of agreement between the 6 different pairs of coders (eg, 2 independent coders for each code) and then taking each of the 6 pairs’ percentage of agreement numbers to calculate the overall group average. Third, team-based analysis and regular meetings were used as a form of peer debriefing, where the team reviewed the codebook, asked questions about the coding process, and provided in-depth feedback and suggestions to ensure the research accurately reported the findings [33].

## Results

### Main Themes Identified

Based on our thematic analysis, 4 main themes were identified: (1) ageism and devaluing the lives of people with dementia, (2) misinformation and false beliefs about dementia and COVID-19, (3) dementia used as an insult for political ridicule, and (4) challenging stigma against dementia.

### Ageism and Devaluing the Lives of People With Dementia

Stigma communication includes processes such as marking some people as different, group labeling to delineate how some people are separate from society, assigning responsibility or blame by making attributions about a group’s actions or way of life, and implying that this group is a peril to society [15,16]. In this sample of tweets, the group labels “poorer quality of life” and “death by COVID-19” were frequently applied to people with dementia, with implications that death by COVID-19 would be a welcome alternative to dying with dementia:

I know two people that have died from Covid. One was 80 with severe dementia and in a nursing home and the other was 35 and topped herself due to lockdown. I know what one was “unnecessary”

Some of the ageist group labeling intersected with negative views of long-term care (LTC) homes:

The life expectancy in LTC (long-term care) is not even 30 months. You're there to die a slow death from Alzheimer's, basically. My neighbour refused treatment for pneumonia for her husband so he wouldn't have to go to the bitter end. Covid is actually a better death than dementia.

In addition, it is important to note the language used when describing dementia in these quotes (ie, “severe”) and in the following quote “full blown”:

That’s one of the really selfish elements of it, because you can guarantee that attitude would change when staring death in the face. Saying that, my granny is 95, in a home, with full blown dementia. Quick Covid death clearly not a disaster there

These tweets present LTC homes or nursing homes as a place where individuals with dementia face a “slow death” and imply limited quality of life. The tweets also suggest that the lived experience of dementia is the same for all older individuals despite having many remaining abilities when first diagnosed and vastly different lived experiences of dementia based on one’s available personal, social, and environmental resources [34]. The tweets do not account for the quality of life as experienced by persons with lived experience of dementia and how the nature of quality of life can change as the disease progresses [35].

In addition to group labeling, the tweets communicated the notion of peril or threat of dementia to the general public. Not only were the lives of people with dementia devalued, but the idea of having dementia as a diagnosis and the possibility that one might die of dementia was reported as a threat (eg, potential diagnosis or death from dementia being the threat) in some of the tweets. For example:

Honestly if I knew I had dementia and was still in charge of myself I'd go out and try and find corona carriers to shake hands with. This coming from someone who watched a close relative existing for 10yrs, NOT LIVING just existing It's horrible. :(

The following tweet has a similar message:

I would consider Covid a blessing if I was in a care home. They don't call pneumonia “the old man's friend” for nothing. A slow dementia death is way way worse.

In both these cases, the tweeters identify as being threatened by dementia, portray dementia as “not living” or a condition with no quality of life, and feel that a COVID-19 death would be preferable. Restricted resources during the pandemic exacerbated the perception that the life of a person with dementia was less valuable:

You have one hospital bed. You can either give to a decrepit 93 yr. old nursing home resident with dementia who’s only realistic COVID outcome is death or a 40 yr. old previously healthy father of 2 young kids who may stand a chance of survival - you choose.

One tweet specifically alluded to the economic threat of a future dementia diagnosis:

. . . I know this is not a popular view - but I would not want to live in a home with dementia and swallow up my kid's inheritance. I'd much, much rather die of COVID than that.

Group labeling reinforces differences between persons with Alzheimer disease and those without Alzheimer disease, which discriminates those with dementia, as it is the end point of stigma.

### Misinformation and False Beliefs About Dementia and COVID-19

Some tweets about COVID-19 and dementia propagated misinformation about dementia. Misinformation about dementia included perpetuating false beliefs that the COVID-19 vaccines (or vaccines in general) cause dementia or labeling COVID-19 as a cause of dementia. The language in these tweets is consistent with the stigma communication model’s processes of responsibility and peril [15,16].

The flu killed more people in 2018 than the corona virus has and we have a flu vaccine that contains Aluminium and Mercury which has been linked to Dementia and Alzheimers

*Did you know; the coronavirus vaccine causes dementia and homosexuality? FAR worse than the virus itself!* *As a mother of 5-oop . . . make that 3, I can't support the corona vaccine! and neither should you! Spread the word across Facebook!*

My friend’s daughter, age 30, has COVID DEMENTIA and COVID COPD - but she is not a statistic unless she dies and many people are not tested.

By perpetuating misinformation about dementia, the end result is that society may view persons with dementia with only negative attributes, and it helps separate “us” from “them.” This view may lead persons with dementia to experience stigma.

### Dementia Used as an Insult for Political Ridicule

Despite use of a filter that referred to the US presidential candidates for the 2020 election by name (Donald Trump and Joe Biden, and synonyms), many tweets remained that were relevant to the theme of insult and political ridicule because these tweets used the term “dementia” in a pejorative manner. The use of dementia as a form of personal attack is an attempt to diminish and devalue others, but it also devalues the lived experiences of those with dementia and their carers. The following examples are tweets that used the term “dementia” with the aim of insulting 1 of the presidential candidates. The stigma communication [15,16] in these tweets is characterized by marking, which refers to an attribute or feature attributed to an individual and “marks” them as different.

To be fair, #DementiaJoe thinks he's running for the US Senate, but I bet your news coverage didn't show that gaffe. Do you really think America is going to elect a cop hating, socialist crook with dementia over the man who built the greatest economy since Reagan, before Covid?

Was unemployment down until covid? Was the economy booming? . . . there’s 2 brutal truths, right there, you’re willing to destroy us all, yourselves included to put a guy with obvious dementia in the WH. That’s insane, and that’s the “brutal truth” of it . . .

More than 60M Americans voted in 2016 to have us led by a malignant narcissist whose dementia was visibly progressing. A shocking percentage of these traitors will vote to re-elect him—and the GOP—even as their redneck family and friends die from COVID-19. America is broken.

Others used the term “dementia” as an insult to imply diminished abilities to perform duties of their job, underscoring how stigma against dementia can include depicting incompetency.

No because the mf has dementia and is unfit too speak in front of a crowd or debate anyone! Covid 19 was a fucking hoax too crash the economy and then you’ve created race wars too deflect off democratic crimes! We know you’re going to rig the election in order to win!!

Others used dementia as an insult to dismiss their target’s viewpoint by suggesting they are cognitively compromised.

Yea, in an alternate reality he might be, but not on this planet in this lifetime. What is it you think is best? He’s blatant racism? The blatant nepotism? Blatant corruption? The way he has allowed Covid to kill our people and destroy the economy? You must have dementia

### Challenging Stigma Against Dementia

One theme emerged in contrast to the others, namely that of challenging the stigma against dementia in association with the COVID-19 pandemic. Tweets condemned the derogatory language, directly critiqued negative behaviors, and voiced concerns that ageism and stigma against persons with dementia were exacerbated during the pandemic. For example, some questioned the lesser value placed on deaths of those with dementia who contracted COVID-19.

It really feels like a portion of the population, we're not supposed to care about their deaths. They're less worthy, or disposable somehow. I imagine someone with dementia or terminal cancer also suffering with end stage severe Covid-19 and my heart breaks.

Some tweets referenced deficits in formal care experienced by persons with dementia and how these have been highlighted by the COVID-19 pandemic. For example, a tweet stated,

It’s a national scandal how dementia patients are treated. COVID-19 took her in the end 😢.

Tweets brought attention to systemic issues around health care access and availability for people with dementia by describing how they deteriorated due to COVID-19 pandemic conditions.

The elder care situation is abhorrent even in the best of times. I witnessed firsthand options in USA for a self pay mother and father w/ dementia and is very dismal. The culture devaluation of elders and economic vulture capitalism has created a living nightmare. Covid amplifies

Further, people wrote tweets as a call to action, directly confronting negative stereotypes of dementia. Some tweets presented accurate facts about dementia to contradict myths or stereotypes. Other tweets asserted a need to change our attitudes and actions with respect to care provision and treatment of older adults with dementia, both generally and specifically regarding the pandemic.

Watched Ross Kemps living with dementia last night. What a great family, dementia isn’t just an old persons illness it can affect anyone at any age. We need to get a hold on this illness, people have hurt long enough. Covid has increased the pain, people need support. @AlzSocNI

#COVID should make us re-examine how we treat the #elderly we will ALL be old one day. We #warehouse people. It’s #shameful and at @SavonixInc we are dedicated to the #dignity of our #elderly #ethics #nursinghomes #dementia

## Discussion

### Principal Findings

During the pandemic, significant social media discourse has focused on COVID-19 and people with dementia [13,17,18]. In this study, we examined Twitter data to understand stigma against people with dementia during the COVID-19 pandemic. Drawing on Smith’s [15,16] model of stigma communication, we identified 4 main themes: ageism and devaluing the lives of people with dementia (eg, group labeling), misinformation and false beliefs about dementia and COVID-19 (eg, peril and responsibility), dementia used as an insult for political ridicule (eg, marking), and challenging stigma against people with dementia. Overall, our study sheds light on stigma against dementia during the COVID-19 pandemic and highlights opportunities for policy and research to address this moving forward.

In our research, we found that Twitter users reported that the lived experience of dementia was the same for all individuals. For example, people with dementia were stereotyped as a homogeneous group of people who were highly vulnerable and at the end stages of their lives. However, research has demonstrated that dementia does not progress in a linear fashion and, most notably, that it varies from person to person [35-37]. Similar stereotypes focusing on frailty and COVID-19 vulnerability among older adults have been found in public health campaigns and traditional news media [5,6,38-40]. These stereotypes serve to perpetuate COVID-19 stigma against older adults and people with dementia. Consequently, COVID-19 policies and public health campaigns should focus on precautions and preventive measures rather than labeling specific population groups.

In several tweets analyzed in this study, there was an assumption that people with dementia were better off dying from COVID-19 than continuing to live with dementia, an assumption that can lead to experienced, perceived, anticipated, or internalized stigma. More specifically, death was described as a welcomed means to end the pain and suffering of people with dementia. This assumption is also embedded throughout the literature prior to the pandemic. For example, in existing studies conducted with caregivers, the death of a person with dementia has been described as the best solution because it ends the person’s pain and suffering due to dementia [41,42]. However, this “solution-based focus” on death is extremely problematic, leaving little scope for developing COVID-19 policies and programs to improve the quality of life for people with dementia. More specifically, how we view and discuss deaths of people with dementia shapes how policymakers, health clinicians, and the public value the lives of people with dementia. Consequently, this type of discourse may influence who is prioritized for treatment in the context of COVID-19 [43].

Although many tweets had stigmatizing content, tweets also challenged stigma against dementia. For example, some tweets provided accurate facts about dementia, highlighting systemic COVID-19 issues faced by people with dementia or directly confronting myths and stereotypes against dementia. This unifying and supportive discourse against stigma and ageism has also been found in other studies [13,17,44,45]. The World Health Organization [21] suggests that sharing accurate information is key to dispelling myths and stereotypes about the disease. Moreover, research suggests that stigma against dementia is related to fear and a lack of understanding about the disease [20,46]. Consequently, there is a growing need for dementia education and awareness campaigns targeted toward digital media [47] and specifically toward Twitter and other social media platforms.

Recent studies show that policymakers, clinicians, and public health officials are increasingly using Twitter to share and gather information on the COVID-19 pandemic [8,48]. However, because Twitter messaging includes opinions as well as information that may be false or inaccurate, precautions must be taken in interpreting the data, especially given the dementia-related stigma identified in our study. Smith et al [49] assert that when stigmatizing discourse is shared with the general public and “influential others,” it becomes a collective norm with policy and practice implications. Specifically, stigma perpetuates misinformation, pejorative language, and patronizing attitudes that can lead to discrimination against people with dementia. Evidently, recent reports highlight discriminatory actions related to the limited provision of medical services, inadequate access to health care information, and restricted access to COVID-19 treatment options and lifesaving supports for people with dementia during the pandemic [50,51]. Accordingly, our findings have practical implications for policymakers and clinicians because they highlight the need for sensitivity to avoid dementia-related stigma, discriminatory actions, and ageist attitudes about COVID-19.

Urgent action is needed to address stigma to improve the quality of life for people with dementia and their care partners [21,22,25,45]. It is essential that people with dementia not be defined by their disease but be instead recognized as individuals with the same human rights as any other person [52,53]. Moreover, people with dementia must be included in critical discussions on COVID-19 programs and policies, especially around the provision of medical services and lifesaving supports. Rather than focusing on stereotypes and stories of risk and suffering, policymakers, clinicians, and the general public must understand that people with dementia are diverse and able to lead meaningful lives. Moving forward, further research is required to identify the contributing factors, implications, and interventions to address stigma against dementia on social media and beyond.

### Limitations

Although our study provides valuable information about perceptions of COVID-19 and stigma against dementia, it is not without limitations. First, our interpretation of each tweet is limited by our lack of knowledge about the author’s tone, intention, and purpose. For example, important details and contextual information (eg, background, culture, confounding factors) may not be included within the 280-character-limit tweet, making the tone and intention of the tweet difficult to assess. To help rectify this issue, each tweet was coded independently by 2 reviewers to reduce the likelihood of misinterpreting the meaning of the tweets. Despite this measure taken in our study, there is still room for interpretative error. Readers of tweets are also limited in their ability to infer tone, intention, and purpose, so there is value is our analysis that assesses and reports on the content of tweets that are being distributed to the public. As such, future research with qualitative interviews or focus groups with people with dementia or their care partners may provide more comprehensive and in-depth information regarding COVID-19 and experienced stigma against dementia.

Second, no sociodemographic or geographic information (eg, ethnicity, age, sex, gender, income, country) of the tweeters was collected in our study and accordingly limits our ability to further evaluate stigma beyond describing the Twitter discourse. For example, because no data were collected on sex or gender, it is difficult to make specific inferences or draw conclusions regarding stigma against dementia in relation to sex or gender during the pandemic. Further research is needed to explore the stigma against dementia in relation to sociodemographic information, including sex and gender.

Third, our data present a snapshot in time because they focused on tweets posted from February 15 to September 7, 2020. Consequently, it is possible that our findings would change if a different time frame was selected, such as during subsequent waves of the pandemic, after the US presidential election had occurred, or after vaccine availability [12]. Further research is necessary to examine longitudinal changes in dementia-related stigma across the duration of the pandemic, across different sociopolitical climates, and across different time frames within the pandemic.

Finally, it is possible we would have discovered more stigma-related tweets if our scraping strategy had not required dementia, COVID-19, and some description of familial or friendly relationship status. We used a reference to US political candidates by name as a filter for the initial study because these were tweets that perpetuated stigma, and although the secondary analysis for the study discovered this theme of stigma perpetuation, it is not clear whether tweets that referred to US political candidates by name were qualitatively different from those that informed the “dementia as an insult for political ridicule theme.”

### Conclusions

During the pandemic, there has been significant social media attention focusing on the increased COVID-19 risks and impacts for people with dementia and their care partners. Unfortunately, much of this discourse has amplified issues of preexisting ageism and stigma against people with dementia. Our study identified 4 themes related to dementia-related stigma and COVID-19, ranging from misinformation and false beliefs to challenging stigma against dementia. Consequently, social media has been used to spread stigma, but it can also be used to challenge these negative beliefs, stereotypes, and false information.

Our findings reveal that dementia education and awareness campaigns are urgently needed to target social media to address stigma. When stigmatizing discourse of dementia is widely shared and consumed amongst the public, it has public health implications. How society talks about dementia shapes how policymakers, clinicians, and the general public value the lives of people with dementia. Stigma perpetuates negative attitudes, patronizing language, and false information that can lead to inequitable access to lifesaving supports and health care services. Thus, COVID-19 policies and public health messages should focus on precautions and preventive measures instead of labeling people with dementia. Consequently, it is essential that people with dementia not be defined by their disease during the pandemic but be instead recognized as diverse individuals who are able to live meaningful lives with the same human rights as any other person.

## Abbreviations

LTC: long-term care

## References

[1] O’Brien K, St-Jean M, Wood P, Willbond S, Phillips O, Currie D, Turcotte M COVID-19 Death Comorbidities in Canada.

[2] Azarpazhooh MR, Amiri A, Morovatdar N, Steinwender S, Rezaei Ardani A, Yassi N, Biller J, Stranges S, Tokazebani Belasi M, Neya SK, Khorram B, Sheikh Andalibi MS, Arsang-Jang S, Mokhber N, Di Napoli M (2020). Correlations between COVID-19 and burden of dementia: an ecological study and review of literature. J Neurol Sci.

[3] Holder K, Reddy PH (2021). The COVID-19 effect on the immune system and mitochondrial dynamics in diabetes, obesity, and dementia. Neuroscientist.

[4] Allen LD, Ayalon L (2021). "It's pure panic": the portrayal of residential care in American newspapers during COVID-19. Gerontologist.

[5] Fraser S, Lagacé M, Bongué B, Ndeye N, Guyot J, Bechard L, Garcia L, Taler V, Adam S, Beaulieu M, Bergeron CD, Boudjemadi V, Desmette D, Donizzetti AR, Éthier S, Garon S, Gillis M, Levasseur M, Lortie-Lussier M, Marier P, Robitaille A, Sawchuk K, Lafontaine C, Tougas F, CCNA Social Inclusion and Stigma Working Group (2020). Ageism and COVID-19: what does our society's response say about us?. Age Ageing.

[6] Morgan T, Wiles J, Williams L, Gott M (2021). COVID-19 and the portrayal of older people in New Zealand news media. J R Soc New Zealand.

[7] Luckett O, Casey M (2021). The Social Organism.

[8] Rosenberg H, Syed S, Rezaie S (2020). The Twitter pandemic: the critical role of Twitter in the dissemination of medical information and misinformation during the COVID-19 pandemic. CJEM.

[9] Carr CT, Hayes RA (2015). Social media: defining, developing, and divining. Atl J Commun.

[10] Goffman E (1963). Stigma: Notes on the Management of Spoiled Identity.

[11] Thornicroft G, Rose D, Kassam A, Sartorius N (2007). Stigma: ignorance, prejudice or discrimination?. Br J Psychiatry.

[12] Allen LD, Odziemczyk IZ, Perek-Białas J, Ayalon L (2021). "We should be at the back of the line": a frame analysis of old age within the distribution order of the COVID-19 vaccine. Gerontologist.

[13] Barrett AE, Michael C, Padavic I (2021). Calculated ageism: generational sacrifice as a response to the COVID-19 pandemic. J Gerontol B Psychol Sci Soc Sci.

[14] World Health Organization Global Report on Ageism.

[15] Smith RA (2014). Testing the model of stigma communication with a factorial experiment in an interpersonal context. Commun Stud.

[16] Smith RA (2007). Language of the lost: an explication of stigma communication. Commun Theory.

[17] Sipocz D, Freeman JD, Elton J (2021). "A toxic trend?": generational conflict and connectivity in twitter discourse under the #BoomerRemover Hashtag. Gerontologist.

[18] Jimenez-Sotomayor MR, Gomez-Moreno C, Soto-Perez-de-Celis E (2020). Coronavirus, ageism, and Twitter: an evaluation of tweets about older adults and COVID-19. J Am Geriatr Soc.

[19] Oscar N, Fox PA, Croucher R, Wernick R, Keune J, Hooker K (2017). Machine learning, sentiment analysis, and tweets: an examination of Alzheimer's disease stigma on Twitter. J Gerontol B Psychol Sci Soc Sci.

[20] Alzheimer Disease International World Alzheimer Report: Attitudes to Dementia.

[21] World Health Organization Overcoming the Stigma of Dementia.

[22] Bethell J, Pringle D, Chambers LW, Cohen C, Commisso E, Cowan K, Fehr P, Laupacis A, Szeto P, McGilton KS (2018). Patient and public involvement in identifying dementia research priorities. J Am Geriatr Soc.

[23] Shah H, Albanese E, Duggan C, Rudan I, Langa KM, Carrillo MC, Chan KY, Joanette Y, Prince M, Rossor M, Saxena S, Snyder HM, Sperling R, Varghese M, Wang H, Wortmann M, Dua T (2016). Research priorities to reduce the global burden of dementia by 2025. Lancet Neurol.

[24] Bacsu J, Mateen FJ, Johnson S, Viger MD, Hackett P (2020). Improving dementia care among family physicians: from stigma to evidence-informed knowledge. Can Geriatr J.

[25] World Health Organization Towards a Dementia Inclusive Society.

[26] Clement J Number of Monthly Active Twitter Users Worldwide from 1st Quarter 2010 to 1st Quarter 2019.

[27] Godfrey M (2015). Qualitative research in age and ageing: enhancing understanding of ageing, health and illness. Age Ageing.

[28] Eysenbach G (2002). Infodemiology: the epidemiology of (mis)information. Am J Med.

[29] Bacsu J, O'Connell ME, Cammer A, Azizi M, Grewal K, Poole L, Green S, Sivananthan S, Spiteri RJ (2021). Using Twitter to understand the COVID-19 experiences of people with dementia: infodemiology study. J Med Internet Res.

[30] Braun V, Clarke V (2006). Using thematic analysis in psychology. Qual Res Psychol.

[31] Birks M, Chapman Y, Francis K (2008). Memoing in qualitative research: probing data and processes. J Res Nurs.

[32] Barry CA, Britten N, Barber N, Bradley C, Stevenson F (1999). Using reflexivity to optimize teamwork in qualitative research. Qual Health Res.

[33] Creswell JW (2007). Qualitative Inquiry and Research Design: Choosing among Five Approaches (2nd ed.).

[34] Clare L, Wu Y, Jones IR, Victor CR, Nelis SM, Martyr A, Quinn C, Litherland R, Pickett JA, Hindle JV, Jones RW, Knapp M, Kopelman MD, Morris RG, Rusted JM, Thom JM, Lamont RA, Henderson C, Rippon I, Hillman A, Matthews FE, IDEAL Study Team (2019). A comprehensive model of factors associated with subjective perceptions of "living well" with dementia: findings from the IDEAL Study. Alzheimer Dis Assoc Disord.

[35] Gallagher R (2019). The lived experience of people with dementia. BCMJ.

[36] Kitwood T (1998). Toward a theory of dementia care: ethics and interaction. J Clin Ethics.

[37] Mitchell G, Agnelli J (2015). Person-centred care for people with dementia: Kitwood reconsidered. Nurs Stand.

[38] Lagacé M, Doucet A, Dangoisse P, Bergeron CD (2021). The "vulnerability" discourse in times of Covid-19: between abandonment and protection of Canadian Francophone older adults. Front Public Health.

[39] Meisner BA (2020). Are you OK, boomer? Intensification of ageism and intergenerational tensions on social media amid COVID-19. Leis Sci.

[40] Rueda J (2021). Ageism in the COVID-19 pandemic: age-based discrimination in triage decisions and beyond. Hist Philos Life Sci.

[41] Hennings J, Froggatt K (2019). The experiences of family caregivers of people with advanced dementia living in nursing homes, with a specific focus on spouses: a narrative literature review. Dementia (London).

[42] Peacock S, Duggleby W, Koop P (2013). The lived experience of family caregivers who provided end-of-life care to persons with advanced dementia. Pall Supp Care.

[43] Apriceno M, Lytle A, Monahan C, Macdonald J, Levy SR (2021). Prioritizing health care and employment resources during COVID-19: roles of benevolent and hostile ageism. Gerontologist.

[44] Danilovich M, Tsay J, Al-Bahrani R, Choudhary A, Agrawal A (2018). #Alzheimer's and dementia. Top Geriatr Rehabil.

[45] Cheng TY, Liu L, Woo BK (2018). Analyzing Twitter as a platform for Alzheimer-related dementia awareness: thematic analyses of tweets. JMIR Aging.

[46] Government of Canada A Dementia Strategy for Canada: Together We Aspire.

[47] Neter E, Chachashvili-Bolotin S, Erlich B, Ifrah K (2021). Benefiting from digital use: prospective association of internet use with knowledge and preventive behaviors related to Alzheimer disease in the Israeli survey of aging. JMIR Aging.

[48] Haman M (2020). The use of Twitter by state leaders and its impact on the public during the COVID-19 pandemic. Heliyon.

[49] Smith RA, Zhu X, Fink EL (2019). Understanding the effects of stigma messages: danger appraisal and message judgments. Health Commun.

[50] Amnesty International Italia Italy: Violations of the Human Rights of Older Residents of Care Homes during COVID-19 Pandemic.

[51] Suárez-González A, Livingston G, Low L Impact and Mortality of COVID-19 on People Living with Dementia: Cross Country Report.

[52] Alzheimer’s Society of Canada Canadian Charter of Rights for People with Dementia.

[53] United Nations Convention on the Rights of Persons with Disabilities.

